# Multiple matings modify the estrous length, the moment of ovulation, and the estradiol and LH patterns in ewes

**DOI:** 10.1590/1984-3143-AR2021-0045

**Published:** 2021-09-24

**Authors:** Juan Pedro Bottino, Raquel Pérez-Clariget, Mariana Garcia Kako Rodriguez, Marcelo Ratto, Rodolfo Ungerfeld

**Affiliations:** 1 Departamento de Biociencias Veterinarias, Facultad de Veterinaria, Universidad de la República, Montevideo, Montevideo, Uruguay; 2 Departamento de Producción Animal y Pasturas, Facultad de Agronomía, Universidad de la República, Montevideo, Montevideo, Uruguay; 3 Departamento de Medicina Veterinária Preventiva e Reprodução Animal, Faculdade de Ciências Agrárias e Veterinárias, Universidade Estadual Paulista “Júlio de Mesquita Filho”, Jaboticabal, São Paulo, Brasil; 4 Instituto de Ciencia Animal, Facultad de Ciencias Veterinarias, Universidad Austral de Chile, Valdivia, Valdivia, Chile

**Keywords:** copula, estrous cycle, follicle, preovulatory LH surge, sexual behavior

## Abstract

In several species, mating reduces the estrous length and advances ovulation. The aim of this study was to determine if multiple matings reduces the estrous length and modifies the moment of ovulation, as well as the estradiol and LH patterns in ewes. The estrous cycle of Corriedale ewes was synchronized, and the onset of receptivity was monitored every 3 h with rams, avoiding mating. At the estrous onset, ewes were assigned to two experimental groups (*n*=10 each): 1) estrous was monitored every 3 h with a ram avoiding mating (group CON), and 2) a ram was allowed to mate and ejaculate once every 3 h (group MAT). The ovaries were scanned with transrectal ultrasonography and blood samples were collected for measuring 17β-estradiol and LH concentrations every 3 h until ovulation. Estrus was shorter in MAT than CON ewes (24.7 ± 1.5 h vs. 30.4 ± 1.5 h, respectively; *P*=0.02); the proportion of animals that ovulated before the end of estrus was greater in CON ewes: (9/10 vs. 3/10, *P*=0.009). The area under the LH curve (AUC) was greater in MAT than CON ewes (36.1 ± 3.5 ng.h^-1^.mL^-1^ vs 24.9 ± 3.5 ng.h^-1^.mL^-1^ *P*=0.03). However, MAT ewes had a lower 17β-estradiol AUC than CON ewes (41.0 ± 4.9 pg.h^-1^.mL^-1^ vs 59.4 ± 4.9 pg.h^-1^.mL^-1^ *P*=0.01). Mating reduced the estrous length, induced a greater secretion of LH but less total 17β-estradiol secreted and, additionally, ovulation occurred more frequently after the end of estrus in mated ewes.

## Introduction

Mating reduces the estrous length and may advance ovulation in several ruminants. As early as 1950, Marion et al. (1950) reported that ovulation occurred earlier in cows that were mated (penetration and ejaculation) during the first 6-8 hours of behavioral estrus than non-mated animals. This is explained by the advancement of the preovulatory discharge of LH, and thus the moment of ovulation by cervical stimulation and mating (Randel et al., 1973). Estrous length is also shorter in does mated by vasectomized bucks than in non-mated females (Romano and Abella, 1997; Romano et al., 2016), regardless of the number of matings (Romano, 1994a). Mating and insemination also shorten the time between the onset of estrus and ovulation in pigs (Signoret et al., 1972; Waberski et al., 1995).

The mechanism by which mating reduces the estrous length and hastens ovulation is still controversial. On one hand, the physical contact of the penis against the vaginal fornix was proposed as the main stimulus in goats (Romano, 1994b; Romano and Benech, 1996), but in camelids, including the llama, a factor present in the male’s seminal plasma, called beta nerve growth factor (βNGF) induces the LH peak (Ratto et. al., 2012).

In ewes, there is a paucity of information on the possible effects of mating on the dynamics of the estrous period. If ewes remain in continuous physical contact with rams, estrous length is reduced (Fletcher and Lindsay, 1971) and the LH surge and ovulation advanced (Lindsay et al., 1975) compared to ewes isolated from males. However, in those studies ewes were also treated with equine Chorionic Gonadotrophin (eCG), so it is not possible to know the effects of mating in natural estrus. Moreover, as the number of matings is extremely uneven among different ewes (Tilbrook and Cameron, 1990), it is possible that while some ewes have been mated several times, while others may have remained unmated in those earlier studies.

The hypothesis of this study was that multiple matings, including penetration and ejaculation, shortens estrous length, advances ovulation, and modifies the 17β-estradiol and LH patterns in ewes. Therefore, the objectives were to compare the estrous length, LH and 17β-estradiol patterns, and the moment of ovulation in mated and unmated estrous ewes.

## Methods

### Animals and general management

The experiment was performed at the Estación Experimental “Bernardo Rosengurt” from the Facultad de Agronomía, Universidad de la República (Cerro Largo, Uruguay, 32° S), during the breeding season (March, autumn in the south hemisphere). The experimental protocol was approved by the Comisión de Ética en el Uso de Animales (CEUA) of the Universidad de la República (UdelaR). Initially, the estrous cycle of 30 multiparous Corriedale ewes was synchronized to ensure the availability of 20 ewes finally included in the study (4 to 6 years old, 51.9 ± 2.2 kg, mean ± SEM). A clinical and gynecological examination and transrectal ultrasonography were performed using a 7.5 MHz linear array transducer coupled to an IUStar monitor (United Imaging, IUStar 160 Vet model, Beijing, China) to determine health and reproductive status 21 days before beginning the study. Ewes continued to graze natural pastures and received rice bran (300 g.animal^-1^.day^-1^) during the period of the study. Four Corriedale rams (4 – 6 years old, 49.8 ± 3.3 kg) were also used in the study. The rams were all sexually experienced and were andrological examined one month before beginning the study.

To reduce the number of animals that were examined simultaneously, the study included two replications (15 ewes initially synchronized in each), beginning the treatments with 5 days of separation between replications. Estrous cycles of all ewes were synchronized using two doses of a PGF-alpha analogue (cloprostenol sodium; 2.53 µg/kg; Ciclase DL, Zoetis, Buenos Aires, Argentina) 14 days apart. Estrous onset was checked in small pens every 3 h beginning 24 h after the second dose, introducing a tethered ram. The ram was allowed to mount the ewes taking extreme care to prevent mating (penis penetration), withdrawing the ram when ewe receptivity was confirmed (when the female remained immobile accepting the mount of the male). In each detection in which there were two or an even number of ewes in estrus, one of them was randomly assigned to one of two experimental groups. In one group, ewes’ receptivity was monitored with the same system every 3 h, determining if the ewe was still in estrus, but preventing mating (group CON, *n*=10, 5 in each replication, 0 matings). The ewes of the other group were mated once with a ram in each detection, every 3 h, confirming the ejaculation observing the intensive perineal contractions and the postejaculatory immobilization (group MAT, n=10, 5 in each replication, 6.4 ± 0.5 matings, mean ± SEM). The detection of estrous receptivity continued for each ewe every 3 h until the ewe was not receptive to further mounting. The rams were changed in every detection and each group using the four rams in both groups.

### Ovarian ultrasound examination

After confirming the receptivity and before allocating each ewe to an experimental group, ovaries were examined by transrectal ultrasound using a 7.5 MHZ linear transducer (United Imaging, IUStar 160 Vet model, Beijing, China), determining the size of the preovulatory follicle every 3 h. Briefly, the animal remained in a standing position, inserting the probe, previously lubricated with carboxymethyl cellulose gel, into the rectum. After locating the uterus, both ovaries were searched for and located, and the diameters of all the follicles greater than 3 mm were measured. Two diameters were measured for each follicle, and the average was calculated. These follicles were measured again in the subsequent moment of estrus detections to determine their regression or ovulation. Only follicles that exceeded 5 mm were considered as possible ovulatory follicles, and ovulation was considered when a follicle greater than 5 mm disappeared. Ultrasounds were performed every 3 h from the time the estrous was detected until 3 h after ovulation.

### Collection of blood samples, and measurement of 17β-estradiol and LH concentrations

After the ultrasound scanning, blood was collected by jugular venipuncture to determine estradiol and LH concentrations. Samples were placed in dry tubes without anti-coagulants, centrifuged at 3000 rpm for 15 min, and serum was separated and stored at – 20 °C until hormonal measurements were performed. Serum LH concentrations were measured using a double-antibody liquid phase radioimmunoassay with ovine LH radioiodinated (LER 1374-A), ovine antibodies CSU-204 and standard ovine LH oLH-S25 (provided by NIADDK, USA) in 200 µL in duplicate, according to the procedure described by Recabarren et al. (1996). The mean intraassay coefficient of variation was 3.6% and the minimum detectable limit of the assay was 0.10 ng/mL. Serum 17β-estradiol concentration was determined using a commercial liquid phase kit DIAsource E2-RIA-CT (DIAsourceInmunoAssays S.A., Louvain-la-Neuve, Belgium). The mean intraassay coefficient of variation was 5.5% and the minimum detectable limit of the assay was 0.01 pg/mL.

### Definitions and statistical analysis

The onset of estrus was considered the mean time between the last time than an ewe did not stand to be mounted by the ram and the first time it was receptive to the ram (Ungerfeld and Rubianes, 1999). Similarly, the end of the estrus was defined as the mean time between the last time that the ewe stands to be mounted and the first in which was not receptive to the ram. The estrous length was considered the time elapsed between the onset and the end of estrus. The time of ovulation was considered the midpoint between the last moment that the preovulatory follicle was observed and the time in which it was not observed again (Roelofs et al., 2004).

The onset of the LH peak was considered as the first value greater than 3 ng/mL and the peak was defined as when concentration reached maximum values (Romano et al., 2018). The area under the curve (AUC) of LH and 17β-estradiol were calculated using the GraphPadPrism Demo (GraphPad Software Inc., San Diego, USA). Duration of the LH peak was defined as the time interval from the onset of the LH surge until it returned to the baseline concentration (time interval when values were 3 ng/mL or less).

Estrous length, LH peak duration, intervals from the onset of estrus to ovulation, from the end of estrus to ovulation, from the onset of estrus to the onset of the LH peak, from the onset of the LH peak to the end of estrus, from the onset of LH peak to ovulation, the AUC of LH and 17β-estradiol, and the LH AUC/17β-estradiol AUC ratio, were compared using a mixed model (SAS University Edition). The model included the treatment as the fixed effect and the replication as a random effect. The frequency of ewes having ovulations before or after the end of estrus was compared with Fisher’s exact probability test. The differences were considered statistically significant when *P*≤0.05, and as tendencies when 0.1≤*P*<0.05.

## Results

In ewes of the MAT group, there was a shorter duration of behavioral estrus (*P=*0.02), and the moment of ovulation in relation to the end of estrus was modified (*P=*0.03) (Table 1). Only single ovulations were detected. The proportion of animals having ovulations before the end of estrus was greater in CON than MAT ewes (9/10 vs 3/10, respectively; *P*=0.009). The intervals from the onset of estrus to ovulation, onset of estrus to onset of the LH peak, the onset of the LH peak to end of estrus, and the onset of the LH peak to ovulation, as well as the LH peak duration, did not differ between groups (Table 1). The LH AUC was greater in ewes of the MAT than CON ewes (*P*=0.03), but the 17β-estradiol AUC was greater in ewes of the CON than MAT ewes (*P*=0.01) (Table 2). The relationship LH/17β-estradiol ratio was greater in ewes of the MAT than CON group (*P*=0.04).

**Table 1:** LSmeans, pooled SEM, and *P* value of different reproductive variables and intervals recorded during the estrous detection period in control (CON) and mated (MAT) ewes. Estrus was monitored every 3h, with a ram that was allowed to mount but preventing penis penetration in CON ewes (*n*=10), or ewes were allowed to mate with a ram with there being a vaginal penetration and ejaculation in MAT ewes (*n*=10).

	**CON**	**MAT**	**Pooled SEM**	***P***
Estrous duration (h)	30.4	24.7	1.5	0.02
LH peak duration (h)	8.9	9.0	1.4	ns
Intervals				
Onset of estrous to ovulation (h)	25.8	26.8	1.6	ns
End of estrous to ovulation (h)	-4.6	2.1	2.0	0.03
Onset of estrous to LH peak (h)	1.5	3.5	2.3	ns
LH peak to end of estrous (h)	22.8	16.6	2.3	ns
LH peak to ovulation (h)	19.4	18.7	2.1	ns

**Table 2:** LSmeans, pooled SEM and *P* value of area under the curve (AUC) of LH and 17β-estradiol (E2) and LH/E2 ratio in control (CON, *n*=10) and mating (MAT, *n*=10) groups during the estrous detection period. In CON group the estrous was monitored, every 3h, with a ram that was allowed to mount but preventing penis penetration while in the ewes of the MAT group estrous was monitored, every 3h, with ram was allowed to mount with penetration and ejaculation occurring.

	**CON**	**MAT**	**Pooled SEM**	***P***
AUC LH (ng^.h^-1.mL^-1)^	24.9	36.1	3.5	0.03
AUC E2 (pg^.h^-1.mL^-1^)	59.4	41.0	4.9	0.01
LH/E2 (ng/pg)	0.5	0.9	0.1	0.04

## Discussion

Multiple matings during the period of sexual receptiveness reduced the length of receptiveness, increased the secretion of LH, increases the LH/E2 ratio, and modified the moment of ovulation in relation to the end of estrus in this study. It is interesting that those differences were observed although unmated ewes were located close to the rams, which also aimed to mate them. In this sense, in goats (Romano et al., 2016; Romano et al., 2018), the presence of the bucks itself modifies the estrous response in unmated females. Therefore, the differences could have been even greater if unmated ewes remained isolated from rams, reinforcing the concept of the high impact that contact with males has in the endocrine and ovarian patterns of the follicular phase. In any case, these results might have important practical implications in programs of artificial insemination (AI) as if ewes in estrus are identified with vasectomized rams, castrated androgen-treated males (wethers treated with testosterone) (Fulkerson et al., 1981), or androgenized ewes (Clarke, 1979), the stimulus, and thus, the moment of ovulation might differ.

Mating shortened the estrous length without modifying the time from estrous onset to ovulation but determining that most mated ewes ovulated after the end of the estrus. There are several possible non-opposed explanations for this effect. First, the vaginal-cervical stimulation induces the secretion of oxytocin (Kendrick et al., 1986), a hormone that suppresses ewes’ receptiveness when infused in the ventromedial nucleus (Kendrick et al., 1993). Mated ewes also secreted more LH, and Wheeler et al. (1975) observed that progesterone concentration increases in coincidence with the LH surge, even before ovulation. As progesterone inhibits the secretion of GnRH in ewes (Goodman et al., 2002), which is the main hormone involved in maintaining the receptiveness in ewes (Caraty et al., 2002), this could also explain the advancement of the end of estrus. To confirm this explanation, it would be necessary to determine if the preovulatory concentration of LH modifies the pattern of progesterone increase during that period.

The lower total amount of 17β-estradiol secreted during this period in MAT ewes did not negatively affect the LH secretion, so there may be other mechanisms stimulating the LH discharge. First, mating may directly stimulate LH secretion by an independent pathway from that of 17β-estradiol action. The seminal plasma of rams also contains β-NGF (Harper et al., 1982; Druart et al., 2013), a protein that induces ovulation in llamas (Ratto et al., 2012; Kershaw-Young et al., 2012). Thus, the physical stimulus and/or the content of β-NGF or other molecules contained in the seminal plasma may directly stimulate the GnRH-LH secretion. It is also possible that the oxytocin released as a consequence of mating (Kendrick et al., 1986) stimulate directly the pituitary release of LH, as happens in the marmoset (O’Byrne et al., 1990), rats, humans, and dogs (Shibusawa et al., 1955), and horses (Alexander et al., 1995). In any case, it is clear that mating induced the secretion of LH by a pathway independent of 17β-estradiol secretion, opening interesting possibilities to study deeply alternatives to handle the moment of ovulation in ewes.

The shortening of the period of behavioral estrus induced by repeated matings may have important implications in the sexual selection process. In polyandric species as sheep, rams compete for access to estrous females (Preston et al., 2001; Ungerfeld and Lacuesta, 2015). However, in competitive breedings, even in wild conditions or in farmed animals, the final individual progeny produced by different rams is influenced by their social behavior, but also by the direct impact of its mating dynamics. For example, the second ram that mates an ewe eliminates an important volume of semen ejaculated by the first ram from the vagina of the ewe (Tilbrook and Pearce, 1986). In promiscuous species there is also sperm competition after semen deposition, reducing the probability of sperm deposited as a result of a specific mating being involved in fertilization (Parker and Birkhead, 2013). Thus, as different rams consistently prefer to mate with the same ewes (Tilbrook and Lindsay, 1987), and mate her repeatedly, the receptive period is shorten reducing the probability of being impregnated by another ram.

## Conclusion

Overall, it was concluded that multiple matings reduce the length of behavioral estrus and modify the moment of ovulation in relation to the end of estrus. Furthermore, it induced a greater secretion of LH even when the ewe had relatively lesser estradiol concentrations. Thus, frequent mating greatly affects the endocrine pattern and the moment of ovulation in ewes.
